# Electrostatics does not dictate the slip-stacked arrangement of aromatic π–π interactions†

**DOI:** 10.1039/d0sc02667k

**Published:** 2020-06-05

**Authors:** Kay Carter-Fenk, John M. Herbert

**Affiliations:** a Department of Chemistry & Biochemistry, The Ohio State University Columbus OH USA herbert@chemistry.ohio-state.edu

## Abstract

Benzene dimer has long been an archetype for π-stacking. According to the Hunter–Sanders model, quadrupolar electrostatics favors an edge-to-face CH⋯π geometry but competes with London dispersion that favors cofacial π-stacking, with a compromise “slip-stacked” structure emerging as the minimum-energy geometry. This model is based on classical electrostatics, however, and neglects charge penetration. A fully quantum-mechanical analysis, presented here, demonstrates that electrostatics actually exerts very little influence on the conformational landscape of (C_6_H_6_)_2_. Electrostatics also cannot explain the slip-stacked arrangement of C_6_H_6_⋯C_6_F_6_, where the sign of the quadrupolar interaction is reversed. Instead, the slip-stacked geometry emerges in both systems due to competition between dispersion and Pauli repulsion, with electrostatics as an ambivalent spectator. This revised interpretation helps to rationalize the persistence of offset π-stacking in larger polycyclic aromatic hydrocarbons and across the highly varied electrostatic environments that characterize π–π interactions in proteins.

## Abstract 2

According to the Hunter–Sanders model, geometries in π–π systems arise from competition between quadrupolar electrostatics (favoring an edge-to-face geometry) and London dispersion (favoring stacking), but this model misrepresents the molecular physics.

## Graphical abstract

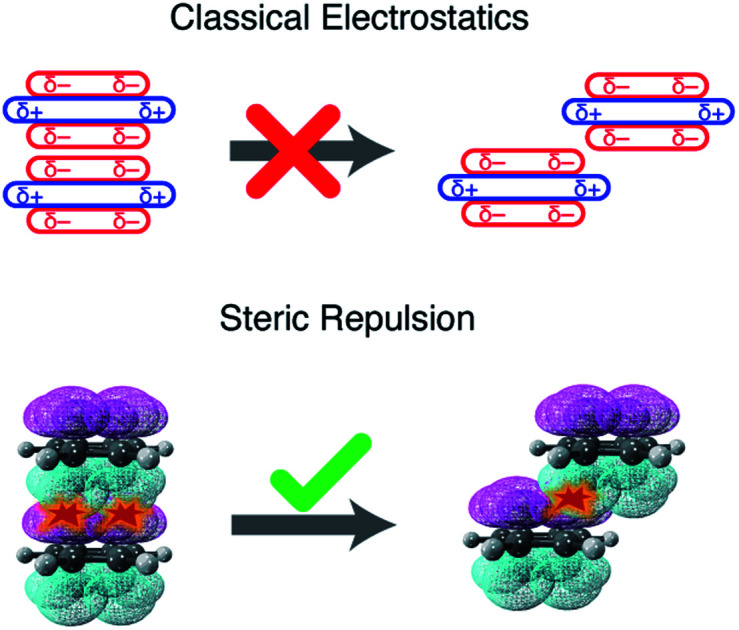

## Introduction

Understanding the factors that govern geometric preferences of π–π interactions is of vital importance in crystal engineering,^1^ with implications as well for understanding protein structure^2–4^ and biological recognition, including drug design.^5^ Benzene dimer has long served as an archetype for understanding the geometric preferences of aromatic π–π interactions,^6–9^ though its emblematic status has occasionally been questioned.^10^

**Fig. 1:**
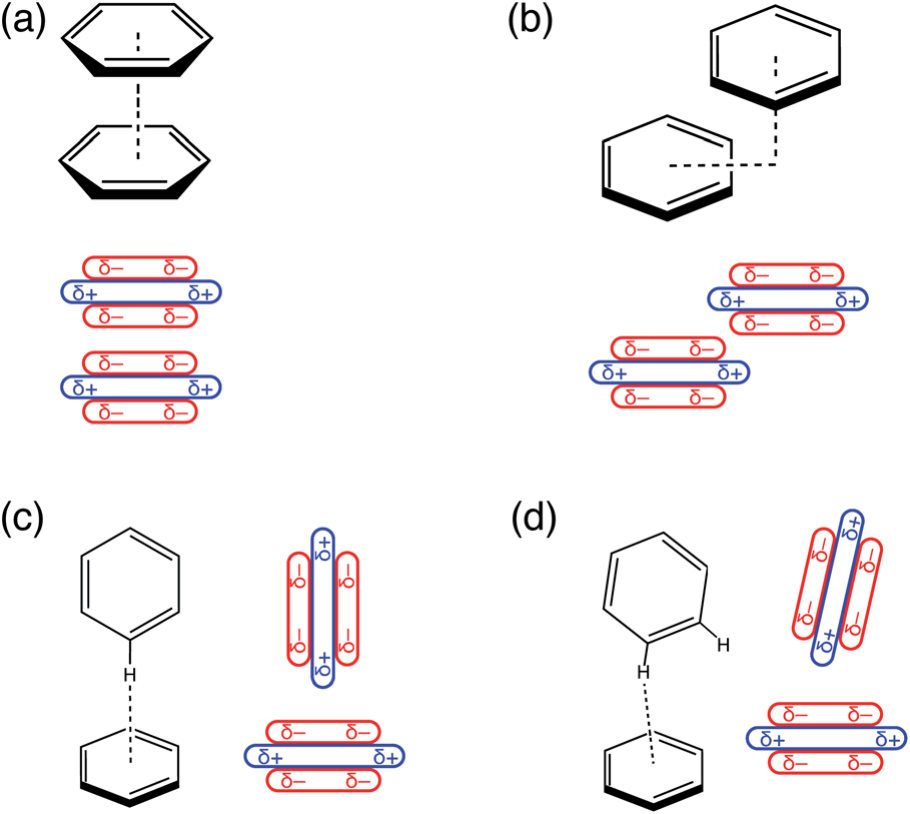
Stationary points on the (C_6_H_6_)_2_ potential energy surface: (a) cofacial π-stacked configuration, (b) “slip-stacked” or “offset-stacked” configuration, (c) canonical T-shaped geometry, and (d) tilted T-shaped geometry. For each structure, a schematic view of the quadrupolar charge distribution is provided.

Relevant conformations of (C_6_H_6_)_2_ include a cofacial “sandwich” geometry (Fig. 1a), a “slip-stacked” or parallel-displaced geometry (Fig. 1b), and a T-shaped isomer characterized by a CH⋯π interaction (Fig. 1c). For (C_6_H_6_)_2_ in the gas phase, the cofacial π-stacking arrangement is an energetic saddle point along a sliding coordinate that leads to the slip-stacked structure,^7^ which is essentially iso-energetic with the T-shaped geometry.^6–9^ The latter is also a saddle point,^8^ with the slightly tilted structure depicted in Fig. 1d emerging as the global minimum.^8^ However, the potential surface is extremely flat along the pendular coordinate that connects the canonical T-shaped and the tilted isomer, which differ by only about 0.2 kcal mol^−1^.^8,9^

In a landmark paper,^11^ Hunter and Sanders developed a simple model to explain these conformational preferences, based on benzene's sizable quadrupole moment.^11–13^ As illustrated in Fig. 1, the Hunter–Sanders model suggests that cofacial π-stacking requires quadrupolar electrostatic repulsion to be compensated by attractive dispersion. In contrast, the T-shaped isomer is stabilized by quadrupolar electrostatics but sacrifices some of the favorable dispersion interaction, because the π clouds of the two monomers are farther apart as compared to the cofacial geometry. The adversarial relationship between electrostatics and dispersion has since emerged as the principle paradigm for interpreting conformational preferences in aromatic systems,^13–21^ and in particular is used to explain the emergence of the slip-stacked configuration in (C_6_H_6_)_2_.^11^

Since the seminal paper by Hunter and Sanders,^11^ additional analysis has begun to erode the simple electrostatic picture proffered by this model. For example, a thorough survey of stationary points on the (C_6_H_6_)_2_ potential surface reveals that electrostatic interactions are actually attractive in the sandwich geometry.^8^ The need for a quantum-mechanical treatment of electrostatics is underscored by studies of substituted benzene dimers by Sherrill and co-workers^22–26^ that illustrate the importance of charge-penetration effects (missing in the quadrupolar electrostatic picture) and demonstrate that electrostatic attraction increases upon substitution, regardless of whether the substituent is electron-withdrawing or electron-donating. Work by these authors,^22,26^ as well as by Wheeler and Houk,^27–32^ demonstrates that the Hunter–Sanders model does not always adequately describe the effects of substitution on aromatic π-stacking energies, and draws attention to the ways in which electrostatic potential maps can sometimes be misleading.^28–32^ However, none of this work directly contests the role of electrostatics in determining geometries of π-stacked complexes. Whereas Grimme notes that the Hunter–Sanders model “overemphasizes” quadrupolar electrostatic interactions,^10^ we will suggest that this model simply gets the molecular physics wrong, and that electrostatics in competition with dispersion is a flawed framework for understanding π-stacking interactions.

Despite many efforts to advance the understanding of π–π interactions beyond the Hunter–Sanders model, there has been no attempt to revisit the physical explanation for the origins of offset π-stacking. The Hunter–Sanders model suggests that π–π interactions should always favor the offset-stacked arrangement over the cofacial geometry, and this fact has been taken to suggest that no special π-stacking interaction actually exists.^33^ Note, however, that the minimum-energy geometry in C_6_H_6_⋯C_6_F_6_ is also parallel-displaced,^34–36^ despite the fact that the C–F bonds reverse the polarity of the charge distribution (relative to that in C_6_H_6_), leading to a quadrupolar electrostatic interaction that is attractive rather than repulsive in the cofacial arrangement. Aromatic heterocycles, whose multipole moments are surely quite different from those of either C_6_H_6_ or C_6_F_6_, also adopt offset-stacked geometries.^37,38^ It has also been suggested that quadrupolar repulsion cannot account for the structures adopted by larger polycyclic aromatics, and that geometries of larger graphene analogues may be better understood *via* Pauli repulsion models.^39^ Below, we show that even in the archetypal case of (C_6_H_6_)_2_, the Hunter–Sanders model fails to capture the essence of π–π interactions. We propose an alternative but equally simple explanation in terms of competition between London dispersion and Pauli repulsion, *i.e.*, a “van der Waals” (vdW) interaction model.

## Methods

We investigate potential energy surfaces for both cofacial and CH⋯π arrangements of (C_6_H_6_)_2_, along a one-dimensional center-to-center sliding coordinate, using energy decomposition analysis (EDA) based on symmetry-adapted perturbation theory (SAPT),^40–43^ the preeminent *ab initio* theory of intermolecular interactions.^44^ Specifically, we employ an “extended” SAPT formalism that includes a variational description of polarization effects,^45–47^ though we also include a *δE*_HF_ correction for polarization^42^ and a many-body dispersion contribution.^48,49^ This approach has been shown to provide accurate results in dispersion-dominated complexes.^49,50^ That said, the same conclusions emerge from more traditional SAPT0/jun-cc-pVDZ calculations, an approach that is known to provide accurate interaction energies for (C_6_H_6_)_2_,^51,52^ as well as from EDA based on absolutely localized molecular orbitals (ALMO-EDA).^53,54^ These additional calculations are presented in Section S1 of the ESI.† All calculations were performed using Q-Chem v. 5.3.^55^

## Results and discussion

In the analysis that follows, we will often group together the electrostatic (“elst”) and induction (“ind”, also known as polarization) components of the interaction energy. This “elst + ind” energy (*E*_elst_ + *E*_ind_) represents the quantum-mechanical Coulomb interaction between polarized charge distributions for the two monomers, without any multipole approximation, and fully accounts for interpenetration of the monomer charge densities. We examine this and other energy components along a parallel-displacement or “sliding” coordinate, in either a parallel or a perpendicular configuration. In the former, the distance between the two molecular planes is fixed at 3.4 Å, which is characteristic of the parallel-displaced local minimum in Fig. 1b and smaller than the 3.8 Å separation that characterizes the cofacial (sandwich) saddle point in Fig. 1a. In the perpendicular orientation, the center of one monomer is displaced at a fixed distance of 5.0 Å (representing the T-shaped saddle point in Fig. 1c) from the plane containing the other monomer.

**Fig. 2:**
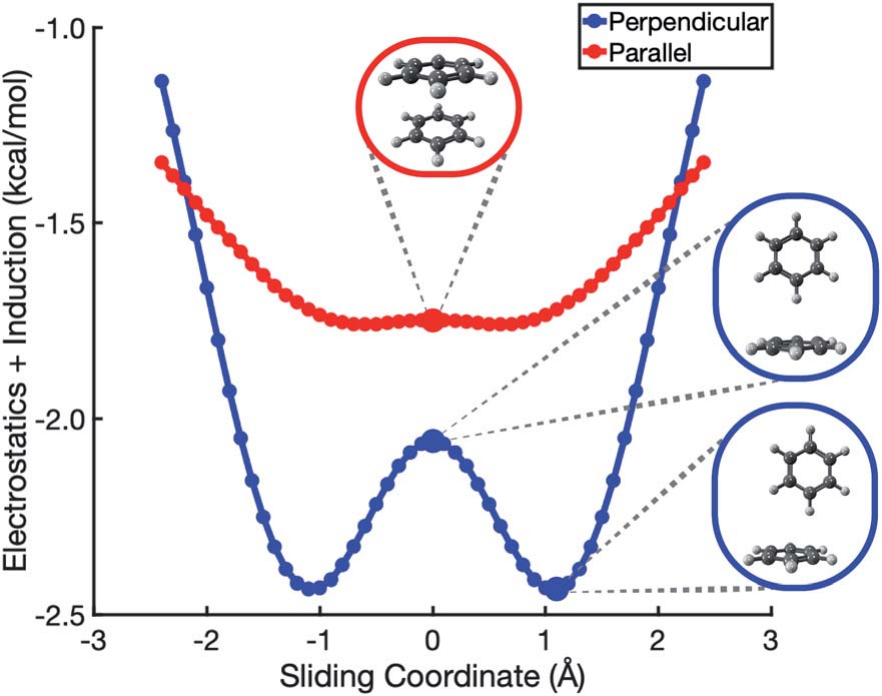
Polarized Coulomb interaction *E*_elst_ + *E*_ind_ along a sliding coordinate in (C_6_H_6_)_2_. The zero-displacement position represents either the sandwich or the T-shaped structure, as shown, with center-to-center monomer distances of 3.4 Å (parallel) and 5.0 Å (perpendicular) at zero displacement.

SAPT-based EDA suggests that the elst + ind interaction energy is nearly flat along the coordinate corresponding to sliding two benzene monomers between cofacial and slip-stacked geometries; see Fig. 2. In the perpendicular configuration of (C_6_H_6_)_2_, the sum of electrostatics and induction exhibits a slight preference (by <0.5 kcal mol^−1^) for a parallel-displaced version of the T-shaped isomer that is not actually a local minimum of the total interaction energy. For both the parallel and perpendicular configurations, these results are precisely opposite to the Hunter–Sanders prediction of a repulsive electrostatic maximum at the sandwich geometry!

This contradiction can be understood in terms of charge penetration that is absent in a model based on classical multipoles. SAPT uses the full electron density to compute both electrostatics and induction, and this has the effect of stabilizing the cofacial structure as compared to the slip-stacked structure; see Fig. S1b.† This stabilization is largely canceled by induction, leading to the relatively flat *E*_elst_ + *E*_ind_ potential in Fig. 2. ALMO-EDA also predicts electrostatic stabilization of the sandwich geometry (Fig. S5†). At larger intermolecular separation, where the density overlap between monomers is negligible, classical electrostatics is recovered and quadrupolar repulsion does become the dominant contribution to electrostatics (see Fig. S3†), but charge penetration is significant at intermolecular separations that typify π–π interactions.^8,24^

**Fig. 3:**
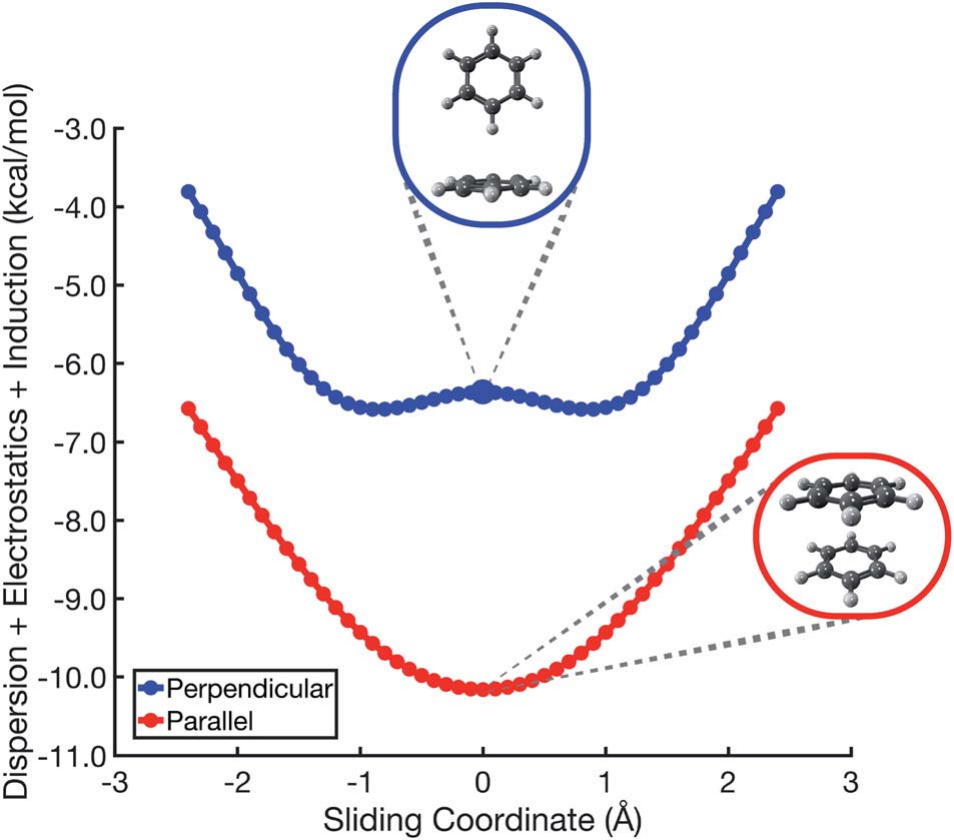
Benzene dimer interaction energies without exchange repulsion, *E*_int_ − *E*_exch_ = *E*_elst_ + *E*_ind_ + *E*_disp_. The parallel-displacement or sliding coordinate is the same as in Fig. 2.

The polarized Coulomb potential (*E*_elst_ + *E*_ind_) alone fails to afford a meaningful saddle point at the sandwich geometry (see Fig. S1b†), and the only energy component that does have a meaningful local maximum in the sandwich configuration is the exchange term (see Fig. S2†). This suggests that it is Pauli repulsion, rather than electrostatics, that provides the driving force towards the slip-stacked arrangement. In fact, when exchange repulsion is removed from the interaction energy, the sandwich geometry emerges as the most stable one, as shown in Fig. 3. With Pauli repulsion removed, the perpendicular configuration demonstrates essentially no preference between the conventional T-shaped isomer and another perpendicular configuration in which the CH⋯π interaction is moved to the edge of the aromatic ring.

**Fig. 4:**
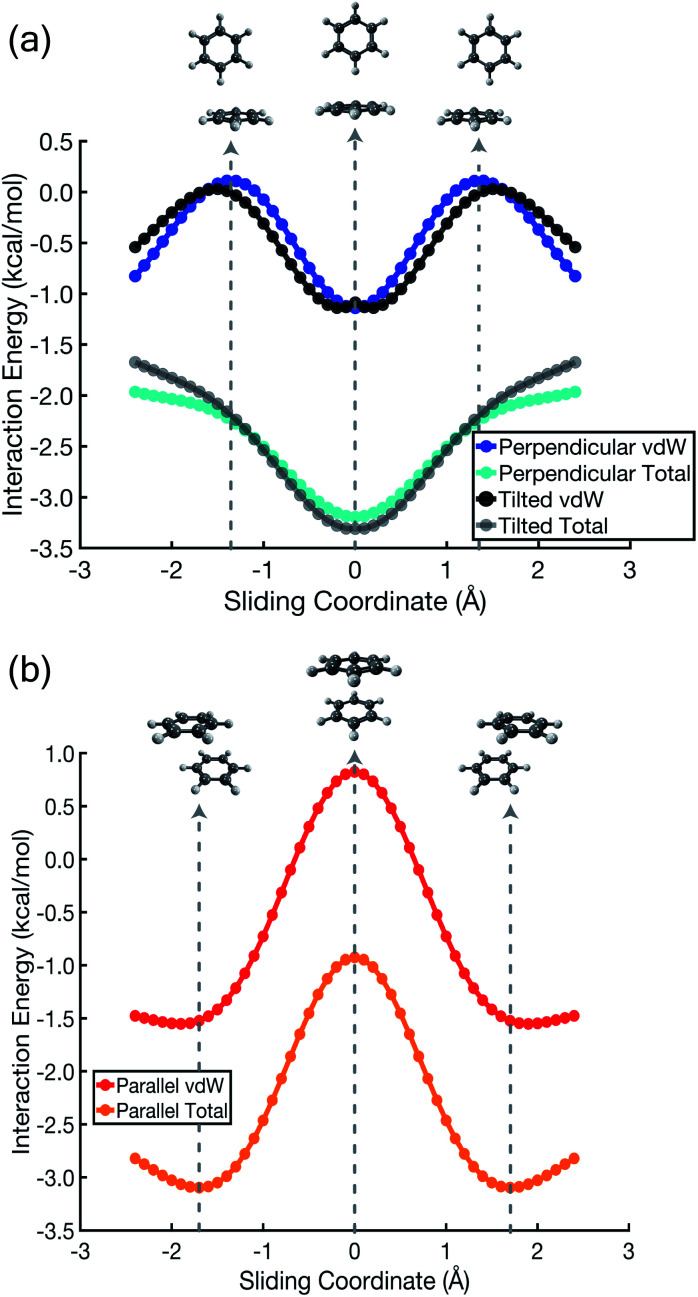
Total interaction energies and vdW interaction potentials (*E*_vdW_ = *E*_exch_ + *E*_disp_), along the parallel-displacement coordinate of (C_6_H_6_)_2_ in (a) perpendicular and (b) parallel orientations. There are two sets of curves in (a), representing the canonical T-shaped isomer (Fig. 1c) and its tilted analogue (Fig. 1d).

A more meaningful picture of π–π interactions can be cultivated by considering a “vdW” interaction potential (*E*_vdW_), which we define to be the sum of Pauli repulsion and dispersion, or in other words the total interaction energy (*E*_int_) minus the polarized Coulomb (*E*_elst_ + *E*_ind_) contribution:*E*_vdW_ = *E*_exch_ + *E*_disp_ = *E*_int_ − *E*_elst_ − *E*_ind_. In comparison to the Coulomb + dispersion potentials in Fig. 3, the vdW potential energy scans in Fig. 4 are much more suggestive of the true stationary points in (C_6_H_6_)_2_, in both its parallel and its perpendicular orientation. The perpendicular orientation (Fig. 4a) exhibits an energy minimum at the T-shaped configuration, not because of attractive electrostatics but rather because dispersion is maximized and Pauli repulsion is minimized. The exchange interaction drives the perpendicular orientation away from “L-shaped” geometries and towards the T-shaped one, because the former exhibits a steric clash between a hydrogen atom on one monomer and the C–C bond density on the other. As noted above, the canonical T-shaped isomer (Fig. 1c) is a saddle point, and a small tilt leads to a minimum that is 0.2 kcal mol^−1^ lower in energy (Fig. 1d).^8^ However, the potential surface along this “pendular” coordinate is quite flat, and sliding potentials for the tilted *versus* T-shaped configurations differ barely at all, as shown in Fig. 4a.

In the cofacial arrangement, (C_6_H_6_)_2_ adopts a slip-stacked geometry that is also a minimum on the vdW surface (Fig. 4b). Because the electron density exhibits local maxima at the nuclei, the sandwich isomer (in which the nuclei of one monomer are situated directly above those of the other monomer) maximizes Pauli repulsion, leading this configuration to be a saddle point on the total interaction potential. The parallel-displaced arrangement reduces this repulsion somewhat, without sacrificing too much of the favorable dispersion interaction. Whereas the elst + ind energy landscape is largely featureless, the vdW landscape exhibits a clear preference for the parallel-displaced and T-shaped geometries.

It should be noted that the cofacial saddle point on the (C_6_H_6_)_2_ potential surface is characterized by a larger intermolecular separation (3.8 Å) as compared to the parallel-displaced minimum (3.4 Å).^8^ For calculations in parallel arrangements, the one-dimensional scans in Fig. 2–4 fix the distance between the two molecular planes at 3.4 Å, which is inside of the repulsive regime for the sandwich geometry. As such, it is worth considering whether this sliding coordinate is a valid proxy for the underlying intermolecular forces. Fundamental to our revised interpretation of these forces is that the nature of the repulsive interaction responsible for offset-stacking is primarily exchange antisymmetry rather than electrostatics, so let us next consider the repulsive forces in detail.

At an intermolecular separation of 3.4 Å, consistent with the parallel-displaced minimum-energy geometry, the vdW potential for the cofacial sandwich structure is strongly repulsive (Fig. 4b) whereas the *E*_elst_ + *E*_ind_ potential is flat (Fig. 2). Pauli repulsion therefore dominates in this close-contact regime, and there are two possible avenues by which the system may ameliorate this repulsion. It can do so either by offsetting the nuclei, leading to the parallel-displaced minimum-energy structure, or else by increasing the intermolecular separation, thereby affording the cofacial saddle point at a separation of 3.8 Å rather than 3.4 Å. This increase in the intermolecular separation reduces charge penetration, making the electrostatics slightly less favorable in the cofacial arrangement as compared to the parallel-offset geometry, but we view this difference in the electrostatics as an effect (driven by Pauli repulsion) rather than a cause. The sandwich structure sacrifices some attractive components of its interaction energy by moving to larger distance in order to minimize Pauli repulsion, whereas the parallel-offset can alleviate Pauli repulsion without significantly increasing the average internuclear separation, thereby affording a lower total energy as compared to the cofacial geometry. Examination of the fixed-separation sliding coordinate is therefore more instructive than simply considering stationary points on the full potential energy surface, because consideration of the repulsive region (*e.g.*, the cofacial arrangement at 3.4 Å) better elucidates the fundamental forces at play. Consideration of stationary points alone might lead one to misattribute the slip-stacking phenomenon to electrostatics and charge penetration.

**Fig. 5:**
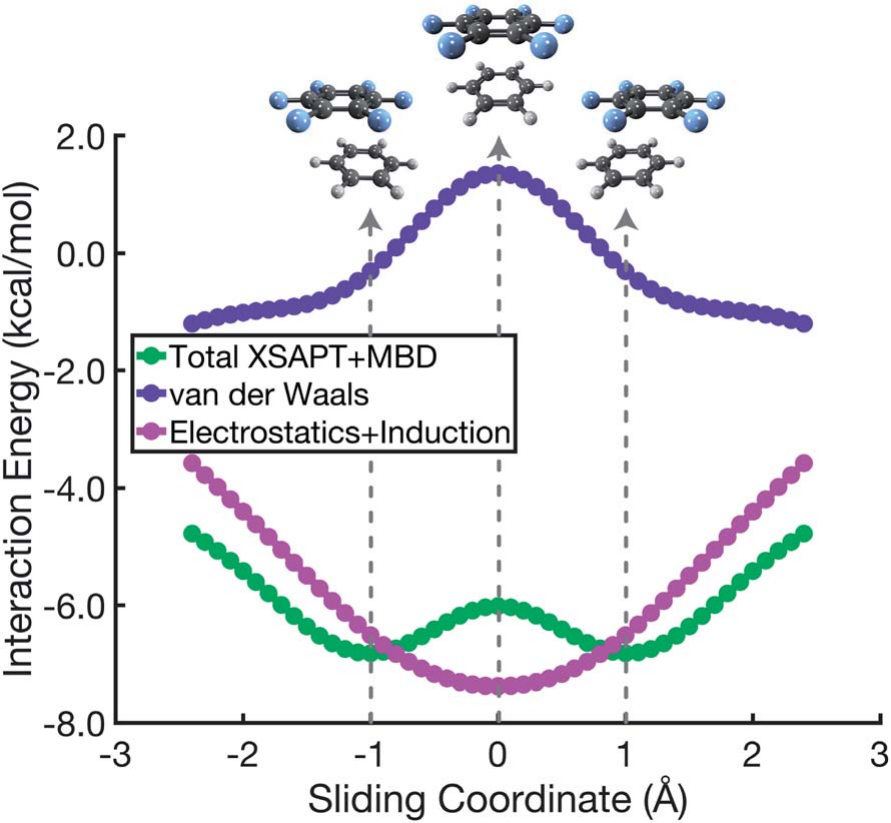
Energy components for a parallel arrangement of C_6_H_6_⋯C_6_F_6_ along the cofacial sliding coordinate. The total interaction energy *E*_int_ = *E*_vdW_ + *E*_elst_ + *E*_ind_ is the sum of vdW and elst + ind components.

The C_6_H_6_⋯C_6_F_6_ system offers an interesting contrast to benzene dimer because its quadrupolar electrostatic interaction is attractive in the cofacial arrangement, which can be understood by switching the signs (*δ*±) in the charge distribution of one monomer in Fig. 1a. Results for benzene dimer already indicate that the Hunter–Sanders model overemphasizes electrostatics, as has been suggested previously.^8,10,25^ Correspondingly, this model predicts a sandwich arrangement for C_6_H_6_⋯C_6_F_6_ as shown in Fig. S6.† SAPT calculations, however, corroborate the notion of an attractive electrostatic interaction that is most significant in the cofacial geometry, but at the same time suggest a parallel-displaced minimum that is ≈1 kcal mol^−1^ lower in total interaction energy. Noting that the total interaction energy*E*_int_ = *E*_vdW_ + *E*_elst_ + *E*_ind_is the sum of vdW and elst + ind components, which are plotted separately in Fig. 5, it is clear that the salient topographical features of the potential surface are inherited from the vdW energy, not from electrostatics. The preference for the parallel-displaced geometry in C_6_H_6_⋯C_6_F_6_ arises for the same reason that it does in (C_6_H_6_)_2_, namely, reducing the density overlap by offsetting the nuclei. Even with its more favorable electrostatics, the energy landscape of cofacial C_6_H_6_⋯C_6_F_6_ is controlled by steric effects.

**Fig. 6:**
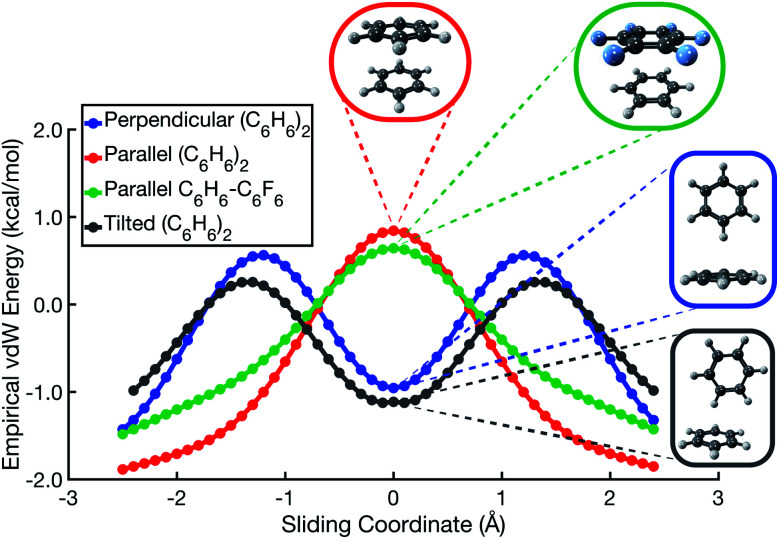
Potential energy scans for parallel, perpendicular, and tilted-perpendicular arrangements of (C_6_H_6_)_2_, along with the parallel configuration of C_6_H_6_⋯C_6_F_6_, obtained from an empirical vdW model that combines an overlap-based repulsive potential with a pairwise-atomic dispersion potential.

In the spirit of preserving a simple model potential capable of qualitative predictions, and to further emphasize the utility in rethinking π–π interactions in terms of vdW forces, we have formulated a two-component model potential. It consists of a repulsive potential proportional to the overlap of atom-centered spheres and an attractive dispersion potential, for which we use the atomic-pairwise *ai*D3 potential.^46^ (For details, consult Section S2 of the ESI†). Parallel-displacement potentials for both (C_6_H_6_)_2_ and C_6_H_6_⋯C_6_F_6_, as predicted by this simple vdW model, are shown in Fig. 6. The model correctly predicts that repulsion is maximized in the on-top sandwich arrangement, and that a T-shaped geometry is favored in the perpendicular edge-to-face configuration. Whereas the Hunter–Sanders model predicts that the cofacial arrangement is a local minimum in C_6_H_6_⋯C_6_F_6_, our vdW model correctly predicts that the sandwich configuration is a saddle point, in agreement with *ab initio* calculations. The vdW model also predicts that the tilted analogue of the T-shaped isomer (Fig. 1d) is slightly lower in energy than the canonical T-shaped structure, again in agreement with *ab initio* results.^8,9^ The model suggests that the tilt angle adopted by the edge-to-face local minimum of (C_6_H_6_)_2_ is driven by gains in dispersion at the expense of a small increase in Pauli repulsion.

**Fig. 7:**
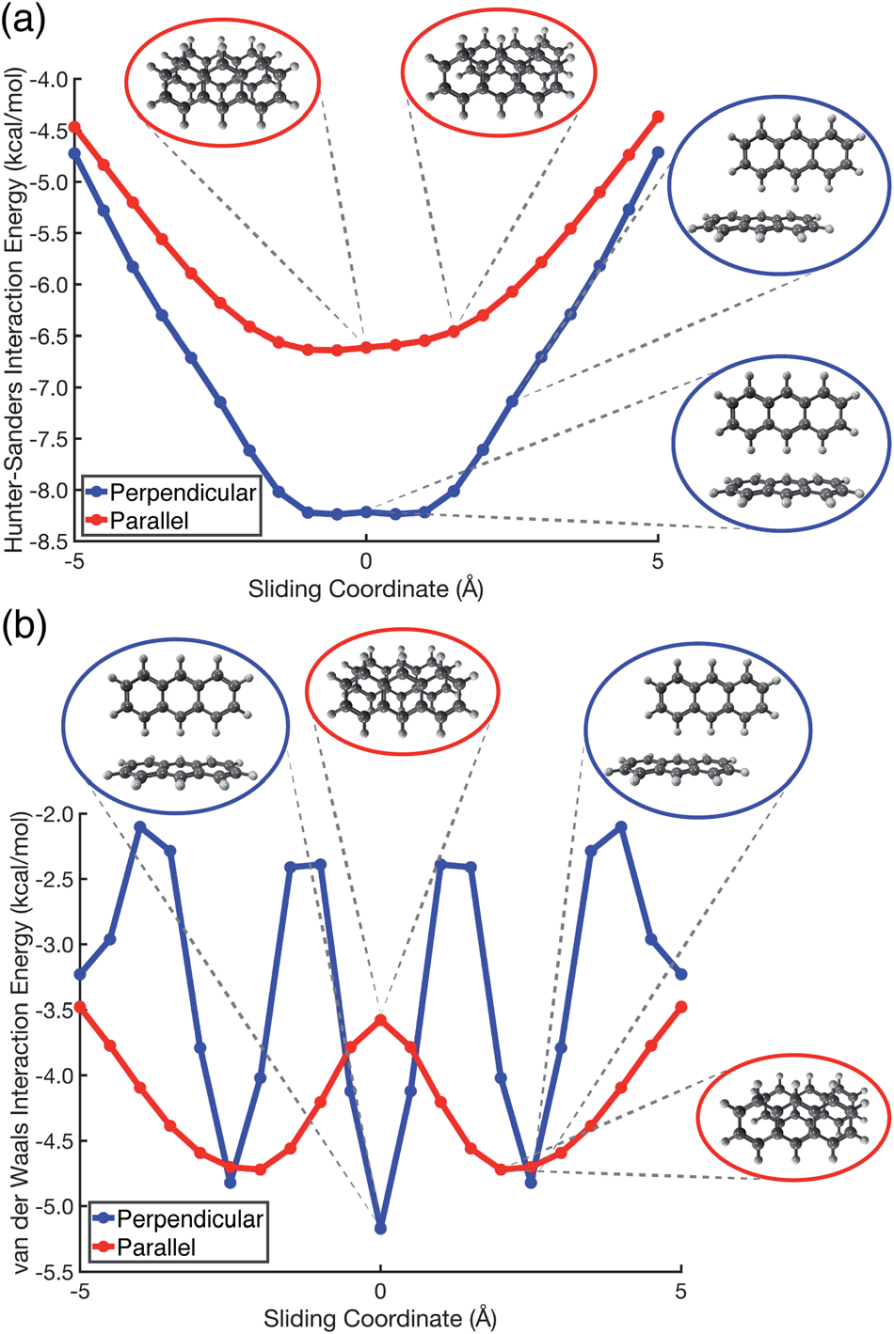
Potential energy scans for parallel displacement of anthracene dimer, as predicted using (a) the conventional Hunter–Sanders model,^11^*versus* (b) the vdW model developed in this work. In (b), the stationary-point geometries coincide with those computed at the TPSS+D3/def2-TZVPP level, which has been recommended for geometry optimizations in large π–π systems.^56^

Pauli repulsion and dispersion are both size-extensive quantities but quadrupolar electrostatics is not, and the latter will saturate in larger polycyclic aromatic systems. In view of this, we examined anthracene dimer using both the Hunter–Sanders model and the new vdW model, for which potential energy surfaces are plotted in Fig. 7 and compared to *ab initio* geometries. The Hunter–Sanders model (Fig. 7a) affords a flat potential for T-shaped (C_14_H_10_)_2_ whereas the vdW model (Fig. 7b) correctly predicts three stationary points corresponding to different CH⋯π motifs. By matching dispersion with commensurate Pauli repulsion, the vdW model correctly predicts that the minimum-energy geometry in the parallel arrangement is slip-stacked, driven by the reduction in Pauli repulsion that comes from offsetting the nuclei. In contrast, the Hunter–Sanders model predicts an offset that is perpendicular to the anthracene ribbon, but no offset along the direction of the ribbon itself, at odds with *ab initio* results. The vdW model is therefore more faithful to *ab initio* geometry optimizations, while retaining the simplicity of the older Hunter–Sanders model. As such, the new model would seem to be a good starting point for constructing more sophisticated empirical force fields whose underlying physics is qualitatively sound.

## Conclusions

Models based on quadrupolar electrostatics, as a means to explain the geometric preferences of (C_6_H_6_)_2_, are textbook material in the field of supramolecular chemistry,^15,16^ despite the abject failure of such models to explain offset-stacking in C_6_H_6_⋯C_6_F_6_, where the sign of the quadrupolar interaction is reversed as compared to benzene dimer. The electrostatics-driven picture of π–π interactions, while valid at sufficiently long range, fundamentally misrepresents the nature of these interactions at typical π-stacking and CH⋯π distances, where charge penetration is significant. More robust conclusions are reached by considering steric repulsion in competition with London dispersion, with electrostatics largely sidelined. A simple vdW interaction potential, introduced here, rationalizes the *ab initio* results and might potentially be developed into a quantitative parameterization for use in force fields.

Armed with a better understanding of the physics that govern π–π interactions, it may be useful to revisit questions regarding the relevance of the “π-stacking” concept,^33^ and the competition between London dispersion and steric repulsion more generally.^57^ Here, we have sought to develop an intuitive model with broad implications for scenarios encountered in biochemistry and crystallography. Our model posits that Pauli repulsion is the dominant force that competes with dispersion in (C_6_H_6_)_2_ and C_6_H_6_⋯C_6_F_6_. Insofar as both forces are always present at short range, this may explain why the slip-stacked geometry (also known as offset-stacking or parallel-displaced π-stacking) emerges as a recurring motif in the π–π interactions found in protein crystal structures,^2,13^ across myriad electrostatic environments, and also in π-stacked complexes involving aromatic heterocycles.^23,37,38^ Electrostatics likely does become relevant once substituents are incorporated (C_6_H_5_X),^22,24–32^ introducing bond dipoles, as well as in solid-state architectures where long-range forces are more important. As an example of the latter, mixtures of C_6_F_6_ with benzene and substituted benzene derivatives are known to organize into columnar π-stacks,^13,14^ with polymorphism that is thought to be controlled by subtle variations in bond-dipole *versus* quadrupolar electrostatics.^58–60^ These phenomena deserve to be considered carefully in light of a new interpretation of π–π interactions.

## Conflicts of interest

J. M. H. serves on the board of directors of Q-Chem Inc.

## Supplementary Material

SC-011-D0SC02667K-s001
