# Supplementary material for: Electrostatics does not dictate the slip-stacked arrangement of aromatic π–π interactions
Source: Chem Sci. 2020 Jun 5;11(26):6758–65. doi: 10.1039/d0sc02667k (PMC8159364; doi:10.1039/d0sc02667k)
Supplement: SC-011-D0SC02667K-s001 [file SC-011-D0SC02667K-s001.pdf]

Electronic Supplementary Material (ESI) for Chemical Science.
This journal is © The Royal Society of Chemistry 2020

Electronic Supplementary Information for "Electrostatics does not dictate the slip-stacked arrangement of aromatic π–π interactions"

Kevin Carter-Fenk and John M. Herbert*

June 1, 2020

Contents

S1 Additional Electronic Structure Calculations **S2**
S1.1 XSAPT+MBD Calculations S2
S1.2 SAPT0 Calculations S4
S1.3 ALMO-EDA Calculations S4

S2 Empirical van der Waals (vdW) Potential **S7**
S2.1 Development of the Model S7
S2.2 Comparison to Hunter-Sanders Model S9

*herbert@chemistry.ohio-state.edu

S1 Additional Electronic Structure Calculations

Geometries of the parallel and perpendicular $(C_6H_6)_2$ isomers were optimized at the TPSS-D3/def2-TZVP level. The equilibrium geometry of the tilted structure was obtained from Ref. 1. The calculations discussed in the main text were all performed at the XSAPT+MBD level,[2] which is described in Section S1.1. To reinforce these conclusions, additional calculations were performed at the more traditional SAPT0 level (Section S1.2),[3] and also using ALMO-EDA (Section S1.3).[4;5]

S1.1 XSAPT+MBD Calculations

Interaction energy calculations reported in the main text were performed using the XSAPT+MBD method.[2] XSAPT is an "extended" version[6–8] of symmetry-adapted perturbation theory (SAPT),[9–11] which includes a self-consistent, variational, many-body charge polarization scheme.[6;12] The many-body dispersion (MBD) scheme developed by Tkatchenko and co-workers[13–15] has been modified for use with SAPT;[2] unlike many empirical dispersion corrections, it represents a valid dispersion interaction at all length scales.

The usual second-order SAPT energy decomposition is

$$E_{\text{int}} = E^{(1)}_{\text{elst}} + E^{(1)}_{\text{exch}} + E^{(2)}_{\text{ind}} + E^{(2)}_{\text{exch-ind}} + E^{(2)}_{\text{disp}} + E^{(2)}_{\text{exch-disp}} \; . \tag{S1}$$

In XSAPT+MBD, the MBD model replaces the SAPT dispersion energy,

$$E_{\text{disp}} = E^{(2)}_{\text{disp}} + E^{(2)}_{\text{exch-disp}} \; . \tag{S2}$$

However the remaining other energy components in Eq. (S1), namely, electrostatics, exchange, and induction, are computed within a SAPT(KS) approach,[16] *i.e.*, using Kohn-Sham (KS) molecular orbitals within the second-order SAPT formalism. The KS wave functions for the monomers were computed at the LRC-ωPBE/def2-TZVPPD level of theory. Range-separation parameters ω were set using the "global density-dependent" (GDD) tuning procedure,[8;17] which affords values $\omega_{\text{GDD}} = 0.340$ bohr^{-1} for C_6H_6 and $\omega_{\text{GDD}} = 0.365$ bohr^{-1} for C_6F_6.

On top of the self-consistent polarization that is included with XSAPT (which is included in $E^{(2)}_{\text{ind}} + E^{(2)}_{\text{exch-ind}}$), a "$\delta E_{\text{HF}}$" correction is included here. Operationally, δE_{HF} requires a Hartree-Fock calculation for the dimer and is used to estimate induction effects beyond second order in perturbation theory.[10] The XSAPT+MBD induction energy is therefore defined as

$$E_{\text{ind}} = E^{(2)}_{\text{ind}} + E^{(2)}_{\text{exch-ind}} + \delta E_{\text{HF}} \; . \tag{S3}$$

However, our conclusions regarding the driving forces for π-stacking are independent of whether δE_{HF} is included in Eq. (S3) or not. Higher-order induction effects serve to flatten out the multipolar potential energy surface (see, *e.g.*, Fig. S4). In effect, higher-order induction cancels the (small) electrostatic stabilization of the sandwich geometry and affords a multipolar energy landscape that is essentially flat along the coordinate that takes the system from the sandwich to the slip-stacked geometry. This flatter multipolar potential surface is consistent with the variational electrostatics obtained in ALMO-EDA calculations when employing classical reference densities, as described in Section S1.3.

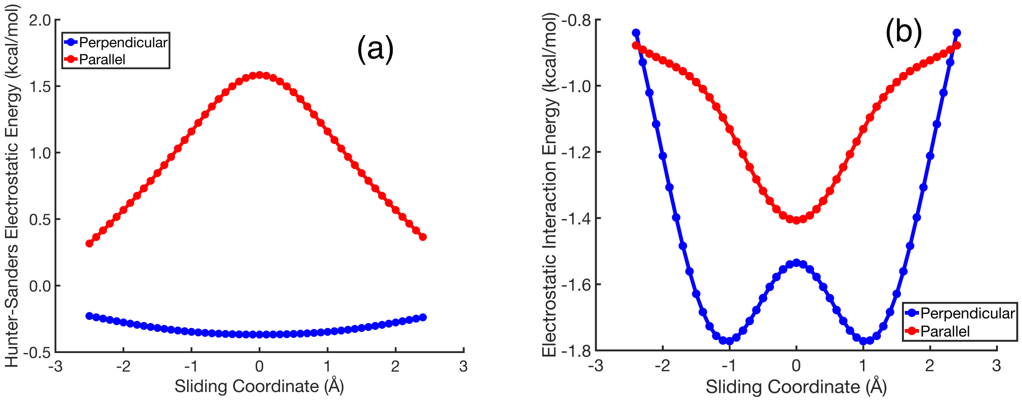

Figure S1: Electrostatic interaction energies along the parallel-displacement coordinate of either the cofacial (parallel orientation) or T-shaped (perpendicular orientation) of $(C_6H_6)_2$. (a) Prediction from the Hunter-Sanders model, *i.e.*, a classical quadrupole–quadrupole interaction. (b) Electrostatic interaction energy $E^{(1)}_{\rm elst}$ obtained from XSAPT+MBD calculations.

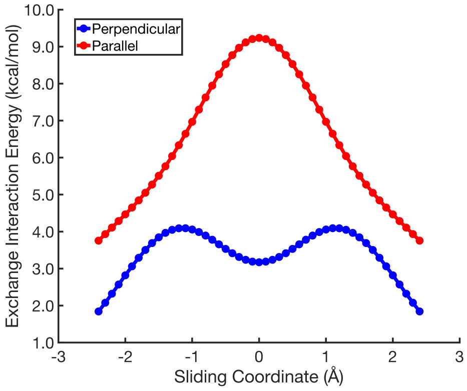

Figure S2: XSAPT+MBD exchange interaction energies ($E^{(1)}_{\rm exch}$) for parallel and perpendicular arrangements $(C_6H_6)_2$ along the parallel-displacement coordinate.

Figure S1 compares XSAPT+MBD electrostatics, defined by the $E^{(1)}_{\rm elst}$ term in Eq. (S1), to the classical quadrupole–quadrupole interaction that is included in the Hunter-Sanders model,[18] for both parallel and perpendicular arrangements of $(C_6H_6)_2$. These results demonstrate that the classical model is simply wrong, qualitatively as well as quantitatively, at least when the center-to-center monomer separation is characteristic of the π-stacked sandwich isomer. In fact it is the exchange term ($E^{(1)}_{\rm exch}$, which is plotted in Fig. S2) that exhibits a local maximum at the sandwich geometry, whereas the electrostatic term exhibits a local *minimum* (Fig. S1b). If the center-to-center separation is increased by 1.1 Å then a classical quadrupolar electrostatic picture does emerge from the SAPT calculations, as shown in Fig. S3. At this separation the monomer densities do not overlap and $E^{(1)}_{\rm elst}$ is indeed repulsive in the the cofacial arrangement.

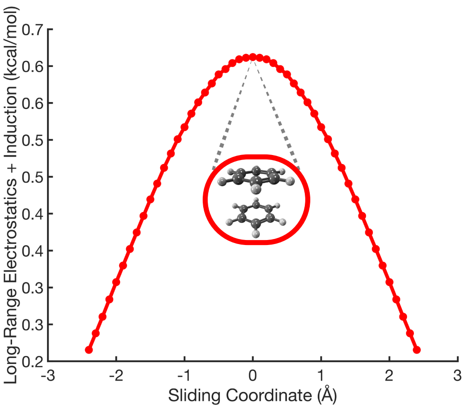

Figure S3: XSAPT+MBD electrostatic interaction energy ($E^{(1)}_{\text{elst}}$) along the parallel-displacement coordinate of cofacial $(C_6H_6)_2$. The center-to-center monomer separation set at $R_0 + 1.1$ Å, where R_0 denotes the equilibrium separation. At this separation, the monomer densities do not interpenetrate and electrostatic interactions are well represented by classical quadrupole moments; the classical quadrupole–quadrupole interaction is repulsive in the cofacial arrangement.

S1.2 SAPT0 Calculations

In order to verify that these results are not artifacts of either SAPT(KS) or the MBD treatment of dispersion, we have performed additional calculations at the SAPT0 level,[3;10] meaning second-order intermolecular perturbation theory applied to Hartree-Fock wave functions for the monomers. Best results with SAPT0 are obtained using the jun-cc-pVDZ basis set,[3] which is used here. SAPT0 energy decompositions for $(C_6H_6)_2$ are plotted in Fig. S4, along the parallel-displacement coordinate of both parallel and perpendicular orientations. The qualitative picture that emerges from these calculations (with or without the δE_{HF} correction) is identical to the one inferred from XSAPT+MBD calculations.

As suggested in the main text, we define the van der Waals (vdW) interaction energy as the sum of Pauli repulsion and London dispersion:

$$E_{\text{vdW}} = E^{(1)}_{\text{exch}} + E^{(2)}_{\text{disp}} + E^{(2)}_{\text{exch-disp}} \, . \tag{S4}$$

SAPT0 results for E_{vdW} are plotted in Fig. S4b, and it can be seen that this partition of the energy successfully captures the topography of the full interaction potential (E_{int}, Fig. S4a), whereas the multipolar potential does not. The latter is defined as

$$E_{\text{mpole}} = E^{(1)}_{\text{elst}} + E^{(2)}_{\text{ind}} + E^{(2)}_{\text{exch-ind}} \, . \tag{S5}$$

This quantity is plotted in Fig. S4d without the δE_{HF} correction, and in Fig. S4c with the δE_{HF} correction included in the induction energy.

S1.3 ALMO-EDA Calculations

The ALMO-EDA method developed by Head-Gordon and co-workers[4;5] is used as a final check on the veracity of the results. These calculations were performed at the ωB97M-V/def2-TZVPPD level of theory and various energy components are plotted in Fig. S5.

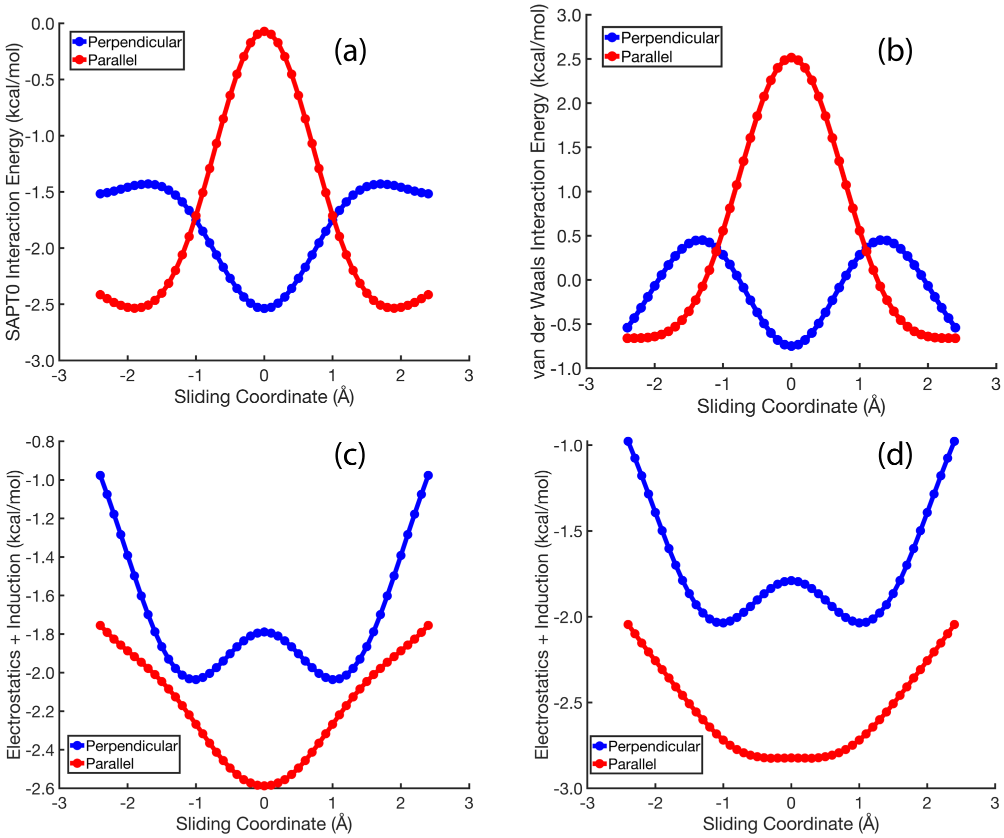

Figure S4: Potential energy surfaces along the parallel-displacement coordinate of $(C_6H_6)_2$, computed at the SAPT0/jun-cc-pVDZ level of theory: (a) total interaction energy, E_{int} in Eq. (S1); (b) van der Waals interaction energy, E_{vdW} in Eq. (S4); (c) multipolar interaction potential, E_{mpole} in Eq. (S5), *without* the δE_{HF} correction; (d) E_{mpole} including the δE_{HF} correction.

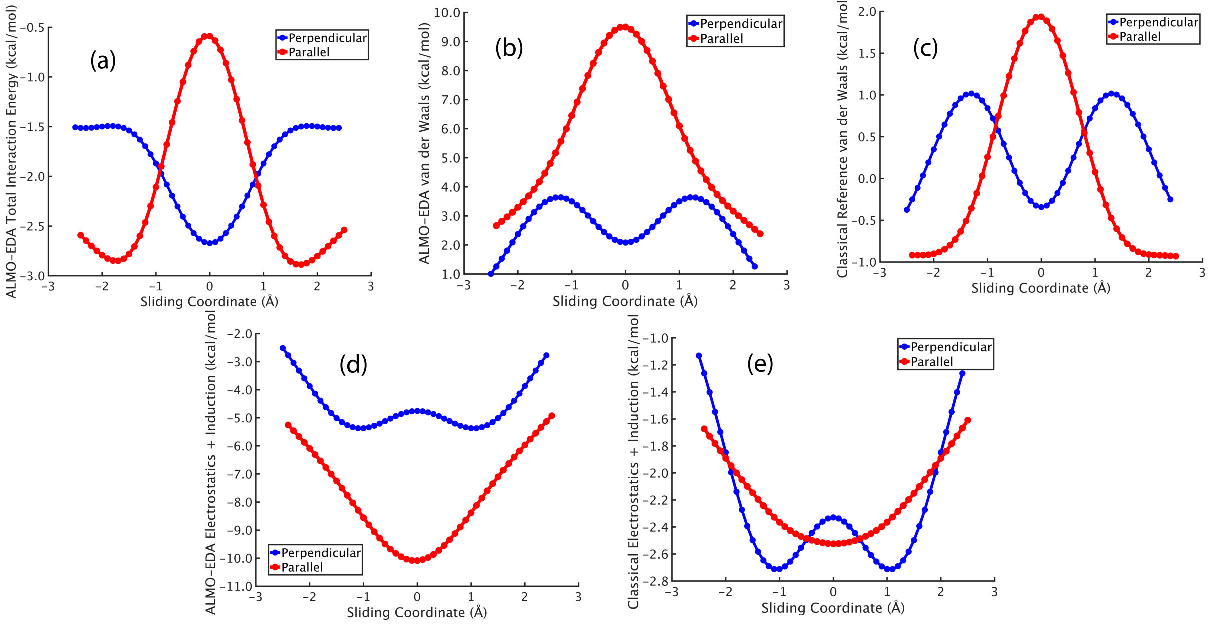

Figure S5: Potential energy surfaces for the sliding coordinate of either cofacial or T-shaped benzene dimer computed using second-generation[5] ALMO-EDA at the ωB97M-V/def2-TZVPPD level of theory, including counterpoise corrections: (a) total interaction energies; (b) vdW interaction energy ($E_{\text{Pauli}} + E_{\text{disp}}$), using an antisymmetrized reference state; (c) vdW interaction energy using a "SAPT-like" (non-antisymmetric) reference state; (d) sum of electrostatics and induction using an antisymmetrized reference state, where induction is taken to be the sum of the ALMO-EDA polarization and charge-transfer energies; (e) electrostatics plus induction (with the latter defined as the sum of polarization and charge-transfer energies), using a SAPT-like reference state that has not been antisymmetrized.

The definition of electrostatics in ALMO-EDA is different from that used in SAPT and warrants some discussion. Within ALMO-EDA, the *classical* electrostatic energy is defined as[4]

$$E_{\text{elst}}^{\text{cls}} = \int\int d\mathbf{r}_1 d\mathbf{r}_2 \, \frac{\rho_A(\mathbf{r}_1)\, \rho_B(\mathbf{r}_2)}{\|\mathbf{r}_1 - \mathbf{r}_2\|} \ , \tag{S6}$$

where $\rho_A(\mathbf{r}_1)$ and $\rho_B(\mathbf{r}_2)$ represent the densities of monomers A and B computed in isolation. This means that the monomer densities are not antisymmetric with respect to exchange of electrons between monomers. Equation (S6) is identical to the definition of electrostatics ($E_{\text{elst}}^{(1)}$ term) used in SAPT. Alternatively, one may define a reference state based on antisymmetrized monomer densities $\tilde{\rho}_A(\mathbf{r})$ and $\tilde{\rho}_B(\mathbf{r})$. The integral that defines the electrostatic interaction is the same one as in Eq. (S6),

$$E_{\text{elst}}^{\text{asym}} = \int\int d\mathbf{r}_1 d\mathbf{r}_2 \, \frac{\tilde{\rho}_A(\mathbf{r}_1)\, \tilde{\rho}_B(\mathbf{r}_2)}{\|\mathbf{r}_1 - \mathbf{r}_2\|} \ , \tag{S7}$$

but the interaction energy defined by Eq. (S7) can be very different from the one in Eq. (S6) because the charge distribution in each monomer has been rearranged to reflect fermion statistics.

ALMO-EDA uses the antisymmetrized definition of electrostatics in Eq. (S7), and the effect of the reference density can be observed by comparing this definition, plotted for $(C_6H_6)_2$ in Fig. S5d, to the classical definition in Eq. (S6) that is plotted in Fig. S5e. In the latter case, the results are in quantitative agreement with both XSAPT+MBD and SAPT0 calculations, since both of the SAPT-based methods use the classical definition at first order. Applying the antisymmetric definition instead, the electrostatic energy becomes much more attractive in the sandwich geometry due to the rearrangement of electron density that is required to maintain antisymmetry. As compared to SAPT-EDA, the ALMO-EDA scheme deviates even further from the Hunter-Sanders model, which predicts electrostatic *repulsion* in the cofacial geometry. Regardless of which reference density is chosen for the analysis, the Hunter-Sanders model does not capture the true nature of the electrostatics in $(C_6H_6)_2$.

S2 Empirical van der Waals (vdW) Potential

S2.1 Development of the Model

In the main text we demonstrate the utility of a simple model potential for predicting the geometries of conjugated ring systems. This vdW potential

$$E_{\text{vdW}} = E_{\text{Pauli}} + E_{\text{disp}} \ , \tag{S8}$$

consists of a Pauli or steric repulsion term and a dispersion term. We take the latter to be the third-generation *ab initio* dispersion potential (*ai*D3) developed previously.[7] It consists of a pairwise sum

$$E_{\text{disp}} = -\sum_{i\in A} \sum_{\substack{j\in B \\ (B\neq A)}} \left[\frac{C_{ij,6}}{R_{ij}^6} f_6(R_{ij}) + \frac{C_{ij,8}}{R_{ij}^8} f_8(R_{ij}) \right] \tag{S9}$$

over atoms i and j in molecules A and B, respectively. The function

$$f_n(R_{ij}) = 1 - \exp(-\beta_{ij} R_{ij}) \sum_{m=0}^{n} \frac{R_{ij}^m}{m!} \tag{S10}$$

is the Tang-Toennies damping function.[19]

Details on the parameterization of *ai*D3 can be found in Ref. 7. Briefly, this potential was fit to dispersion energies computed at the SAPT2+ or SAPT2+(3) level,[3;10] for a test set of small dimers at various intermolecular separations. The *ai*D3 potential therefore represents genuine dispersion at all length scales, unlike many empirical "+D" corrections used in dispersion-corrected DFT.[20] (Among other issues, the latter methods have a double-counting problem for middle-range dispersion,[21] whereas all energy components in SAPT are well-defined and separable, and there is no double-counting.)

To construct E_{Pauli}, we first note that Pauli repulsion can be approximated using the overlap of molecular densities.[22;23] In the interest of having a simple model potential we do not compute the overlap of genuine electron densities but instead model it as the overlap between atom-centered spherical Gaussian functions that stand in for the spatial extent of the atomic densities. The widths σ_i of these Gaussian functions are set equal to scaled atomic radii $\eta R_{\text{vdW},i}$, where η is the scaling factor and $R_{\text{vdW},i}$ is taken from Bondi's set of vdW radii.[24] Setting the size of atom i is accomplished by calculating the variance of a normalized Gaussian,

$$\langle \sigma_i^2 \rangle = \sqrt{\frac{2\alpha_i}{\pi}} \int_{-\infty}^{\infty} x^2 e^{-2\alpha_i x^2} dx = \frac{1}{4\alpha_i} \ . \tag{S11}$$

Setting $\langle \sigma_i^2 \rangle^{1/2} = \eta R_{\text{vdW},i}$ and solving for the Gaussian exponent affords

$$\alpha_i = \frac{1}{4(\eta R_{\text{vdW},i})^2} \ . \tag{S12}$$

The parameter η scales $R_{\text{vdW},i}$ to account for atomic size variations induced by the molecular environment. As we are interested only in qualitative results, we use a uniform-scaling approximation and set $\eta = 0.36$. (This leads to atomic radii that are smaller than Bondi's vdW radii, largely because the interaction is undamped in this simple model. We note that the MBD model uses atomic radii smaller than Bondi's, despite the use of a damping function.[13–15]) We use an empirical description of the Pauli repulsion that is consistent with the semiclassical nuclear de-screening expression used elsewhere,[25]

$$E_{\text{Pauli}} = \sum_{i \in A} \sum_{\substack{j \in B \\ (B \neq A)}} \frac{S_{ij}^2}{R_{ij}} Z_i Z_j \tag{S13}$$

where Z_n are the atomic numbers. This expression can be understood as the classical electrostatic repulsion of the nuclei modulated by the overlap of the electron clouds. The overlap of vdW spheres follows from the spherical Gaussian overlap formula

$$S_{ij} = e^{-\alpha\beta \|\mathbf{A}-\mathbf{B}\|^2/(\alpha+\beta)} \ , \tag{S14}$$

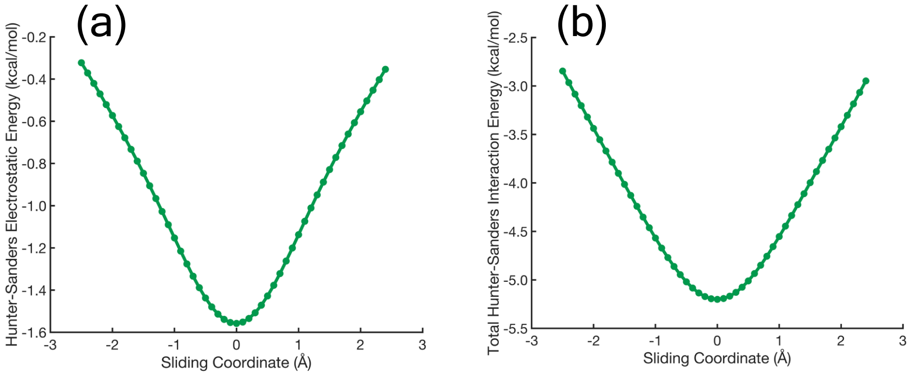

Figure S6: Results from the Hunter-Sanders model for parallel displacement of $C_6H_6 \cdots C_6F_6$, with $x = 0$ corresponding to the sandwich geometry: (a) quadrupolar electrostatic interaction ($E_{\rm elst}$) only, and (b) full Hunter-Sanders potential, $E_{\rm elst} + E_{\rm disp}$. The point charges $\delta\pm$ used to compute $E_{\rm elst}$ were taken to be equal and opposite those used by Hunter and Sanders for $(C_6H_6)_2$.[18]

with α and β being the Gaussian exponents for atoms centered at **A** and **B**, respectively. Clearly, Eq. (S13) breaks down in the limit of large overlap due to the R_{ij}^{-1} dependence. In order to avoid divergence of Eq. (S13) without resorting to a damping function, we apply a "weak overlap approximation" and consider only inter-planar distances > 3.4 Å in accordance with the original Hunter-Sanders model.[18] If an inter-planar distance is naturally less than 3.4 Å, we shift the inter-planar distance to be equal to 3.5 Å. For example, $C_6H_6 \cdots C_6F_6$ has an equilibrium inter-planar separation of just 3.2 Å, so we simply increase the inter-planar distance to match that of benzene dimer (3.5 Å) for the application of this model potential. Perpendicular geometries are left at inter-ring distance found in T-shaped benzene dimer.

S2.2 Comparison to Hunter-Sanders Model

A Hunter-Sanders-type model for $C_6H_6 \cdots C_6F_6$ was obtained by reversing the signs of the point charges $\delta\pm$ relative to those suggested for $(C_6H_6)_2$ in the original work of Hunter and Sanders.[18] As shown in Fig. S6, both the quadrupolar electrostatic component of this model ($E_{\rm elst}$) as well as the dispersion component ($E_{\rm disp}$) drive the system towards a cofacial sandwich geometry, whereas the true minimum-energy geometry obtained from *ab initio* calculations is offset-stacked.[26;27] This behavior apparently cannot be captured without the explicit introduction of a short-range repulsive term, which is present in our vdW model but not in the Hunter-Sanders model.

Figure 7 compares results of the Hunter-Sanders model and the vdW model for parallel and perpendicular orientations of anthracene dimer. In agreement with *ab initio* calculations (TPSS-D3/def2-TZVPP), the vdW model predicts that the minimum-energy geometry in the cofacial arrangement involves displacement along the direction of the long axis of the anthracene molecule. No such displacement is predicted by the Hunter-Sanders model.

References

[1] O. Bludský, M. Rubeš, P. Soldán, and P. Nachtigall, Investigation of the benzene-dimer potential energy surface: Dft/ccsd(t) correction scheme, *J. Phys. Chem.*, 2008, **128**, 114102.

[2] K. Carter-Fenk, K. U. Lao, K.-Y. Liu, and J. M. Herbert, Accurate and efficient *ab initio* calculations for supramolecular complexes: Symmetry-adapted perturbation theory with many-body dispersion, *J. Phys. Chem. Lett.*, 2019, **10**, 2706–2714.

[3] T. M. Parker, L. A. Burns, R. M. Parrish, A. G. Ryno, and C. D. Sherrill, Levels of symmetry adapted perturbation theory (SAPT). I. Efficiency and performance for interaction energies, *J. Chem. Phys.*, 2014, **140**, 094106:1–16.

[4] P. R. Horn, Y. Mao, and M. Head-Gordon, Defining the contributions of permanent electrostatics, Pauli repulsion, and dispersion in density functional theory calculations of intermolecular interaction energies, *J. Chem. Phys.*, 2016, **144**, 114107:1–13.

[5] P. R. Horn, Y. Mao, and M. Head-Gordon, Probing non-covalent interactions with a second generation energy decomposition analysis using absolutely localized molecular orbitals, *Phys. Chem. Chem. Phys.*, 2016, **18**, 23067–23079.

[6] J. M. Herbert, L. D. Jacobson, K. U. Lao, and M. A. Rohrdanz, Rapid computation of intermolecular interactions in molecular and ionic clusters: Self-consistent polarization plus symmetry-adapted perturbation theory, *Phys. Chem. Chem. Phys.*, 2012, **14**, 7679–7699.

[7] K. U. Lao and J. M. Herbert, Accurate and efficient quantum chemistry calculations of noncovalent interactions in many-body systems: The XSAPT family of methods, *J. Phys. Chem. A*, 2015, **119**, 235–253.

[8] K. U. Lao and J. M. Herbert, Atomic orbital implementation of extended symmetry-adapted perturbation theory (XSAPT) and benchmark calculations for large supramolecular complexes, *J. Chem. Theory Comput.*, 2018, **14**, 2955–2978.

[9] K. Szalewicz, Symmetry-adapted perturbation theory of intermolecular forces, *WIREs Comput. Mol. Sci.*, 2012, **2**, 254–272.

[10] E. G. Hohenstein and C. D. Sherrill, Wavefunction methods for noncovalent interactions, *WIREs Comput. Mol. Sci.*, 2012, **2**, 304–326.

[11] G. Jansen, Symmetry-adapted perturbation theory based on density functional theory for noncovalent interactions, *WIREs Comput. Mol. Sci.*, 2014, **4**, 127–144.

[12] K.-Y. Liu, K. Carter-Fenk, and J. M. Herbert, Self-consistent charge embedding at very low cost, with application to symmetry-adapted perturbation theory, *J. Chem. Phys.*, 2019, **151**, 031102:1–7.

[13] A. Tkatchenko, R. A. DiStasio Jr., R. Car, and M. Scheffler, Accurate and efficient method for many-body van der Waals interactions, *Phys. Rev. Lett.*, 2012, **108**, 236402:1–5.

[14] A. Ambrosetti, A. M. Reilly, R. A. DiStasio Jr., and A. Tkatchenko, Long-range correlation energy calculated from coupled atomic response functions, *J. Chem. Phys.*, 2014, **140**, 18A508:1–14.

[15] J. Hermann, R. A. DiStasio Jr., and A. Tkatchenko, First-principles models for van der Waals interactions in molecules and materials: Concepts, theory, and applications, *Chem. Rev.*, 2017, **117**, 4714–4758.

[16] K. U. Lao and J. M. Herbert, Symmetry-adapted perturbation theory with Kohn-Sham orbitals using non-empirically tuned, long-range-corrected density functionals, *J. Chem. Phys.*, 2014, **140**, 044108:1–8.

[17] M. Modrzejewski, L. Rajchel, G. Chalasinski, and M. M. Szczesniak, Density-dependent onset of the long-range exchange: A key to donor–acceptor properties, *J. Phys. Chem. A*, 2013, **117**, 11580–11586.

[18] C. A. Hunter and J. K. M. Sanders, The nature of π–π interactions, *J. Am. Chem. Soc.*, 1990, **112**, 5525–5534.

[19] K. T. Tang and J. P. Toennies, An improved simple model for the van der Waals potential based on universal damping functions for the dispersion coefficients, *J. Chem. Phys.*, 1984, **80**, 3726–3741.

[20] S. Grimme, Semiempirical GGA-type density functional constructed with a long-range dispersion correction, *J. Comput. Chem.*, 2006, **27**, 1787–1799.

[21] S. Grimme, Density functional theory with London dispersion corrections, *WIREs Comput. Mol. Sci.*, 2011, **1**, 211–228.

[22] R. J. Wheatley and S. L. Price, An overlap model for estimating the anisotropy of repulsion, *Mol. Phys.*, 1990, **69**, 507–533.

[23] J. H. Jensen and M. S. Gordon, An approximate formula for the intermolecular Pauli repulsion between closed shell molecules, *Mol. Phys.*, 1996, **89**, 1313–1325.

[24] A. Bondi, Van der Waals volumes and radii, *J. Phys. Chem.*, 1964, **68**, 441–451.

[25] J. A. Rackers and J. W. Ponder, Classical Pauli repulsion: An anisotropic, atomic multipole model, *J. Chem. Phys.*, 2019, **150**, 084104:1–22.

[26] B. W. Gung and J. C. Amicangelo, Substituent effects in C_6F_6–C_6H_5X stacking interactions, *J. Org. Chem*, 2006, **71**, 9261–9270.

[27] S. Tsuzuki, T. Uchimaru, and M. Mikami, Intermolecular interaction between hexafluorobenzene and benzene: Ab initio calculations including CCSD(T) level electron correlation correction, *J. Phys. Chem. A*, 2006, **110**, 2027–2033.
